# Complementary Roles in Cancer Prevention: Protease Inhibitor Makes the Cancer Preventive Peptide Lunasin Bioavailable

**DOI:** 10.1371/journal.pone.0008890

**Published:** 2010-01-26

**Authors:** Chia-Chien Hsieh, Blanca Hernández-Ledesma, Hyun Jin Jeong, Jae Ho Park, Ben O. de Lumen

**Affiliations:** 1 Department of Nutritional Sciences and Toxicology, University of California, Berkeley, California, United States of America; 2 Plant Resources and Environment, Andong National University, Andong, Korea; Health Canada, Canada

## Abstract

**Background:**

The lower incidence of breast cancer among Asian women compared with Western countries has been partly attributed to soy in the Asian diet, leading to efforts to identify the bioactive components that are responsible. Soy Bowman Birk Inhibitor Concentrate (BBIC) is a known cancer preventive agent now in human clinical trials.

**Methodology/Principal Findings:**

The objectives of this work are to establish the presence and delineate the in vitro activity of lunasin and BBI found in BBIC, and study their bioavailability after oral administration to mice and rats. We report that lunasin and BBI are the two main bioactive ingredients of BBIC based on inhibition of foci formation, lunasin being more efficacious than BBI on an equimolar basis. BBI and soy Kunitz Trypsin Inhibitor protect lunasin from *in vitro* digestion with pancreatin. Oral administration of ^3^H-labeled lunasin with lunasin-enriched soy results in 30% of the peptide reaching target tissues in an intact and bioactive form. In a xenograft model of nude mice transplanted with human breast cancer MDA-MB-231 cells, intraperitoneal injections of lunasin, at 20 mg/kg and 4 mg/kg body weight, decrease tumor incidence by 49% and 33%, respectively, compared with the vehicle-treated group. In contrast, injection with BBI at 20 mg/kg body weight shows no effect on tumor incidence. Tumor generation is significantly reduced with the two doses of lunasin, while BBI is ineffective. Lunasin inhibits cell proliferation and induces cell death in the breast tumor sections.

**Conclusions/Significance:**

We conclude that lunasin is actually the bioactive cancer preventive agent in BBIC, and BBI simply protects lunasin from digestion when soybean and other seed foods are eaten by humans.

## Introduction

Breast cancer is the most common malignant tumor among women and the leading causes of death of women in Western countries [1]. In contrast, breast cancer incidence in most Asian countries is approximately 10% that of the general population of the USA and Europe [2]. Of all environmental factors known to influence breast cancer, diet appears to be one of the most significant. Asian diets are rich in soybean products containing factors that have been found to provide important protection against initiation, promotion and/or progression of breast cancer in animal models [3]. In 1980, Troll and coworkers suggested the possibility that soy protein might have a role in preventing breast cancer in irradiated rats [4]. Animal experiments carried out during the last decade have confirmed the breast cancer preventive role of soy protein [2], [3]. In particular, bioactive peptides isolated from soybeans, such as lunasin and the Bowman-Birk protease inhibitor (BBI) are now being intensively studied as cancer chemopreventive agents.

Lunasin is a novel peptide initially identified in soybean [5] and subsequently, isolated in wheat, barley and other seeds [6]–[9]. It is a 43-amino acid peptide which efficacy has been demonstrated in mammalian cells against chemical carcinogens and viral oncogenes [10], [11]. The first mouse model confirmed the chemopreventive activity of lunasin against skin cancer induced by a chemical carcinogen [10]. These results suggest that lunasin may exert a promising role as preventive agent against other types of cancer, such as breast cancer.

BBI is a polypeptide of 71 amino acids with the ability to inhibit the serine proteases trypsin and chymotrypsin. The trypsin inhibitory site of BBI has been associated with negative effects on bioavailability of dietary proteins, whereas the chymotrypsin site has been implicated in cancer chemopreventive effects [12], [13]. The high cost of BBI's purification process has made necessary the use of an impure form of BBI called BBI concentrate (BBIC) that has been reported to exert chemopreventive activity against different types of cancer induced by chemical carcinogens and radiations [14]. These studies consider that BBI is the main component responsible for BBIC's chemopreventive activity, without evaluating the contribution of other peptides contained in the BBIC on its cancer preventive activity.

Oral administration has been recognized as a plausible and cost-effective approach to reduce cancer morbidity and mortality by inhibiting precancerous events before the occurrence of clinical disease [15]. Since lunasin and BBI are peptides, it is crucial to establish whether they, once orally ingested, survive digestion and get absorbed, reaching the target tissues and organs in an intact and bioactive state. Park and coworkers carried out *in vitro* studies demonstrating the role of BBI in protecting lunasin from digestion when soy protein was orally consumed [16]. However, there are no *in vivo* studies examining the role of BBI in protecting lunasin from digestion in the gastrointestinal tract of animals.

The aims of this work are to evaluate the presence and *in vitro* activity of lunasin and BBI contained in BBIC and study their bioavailability after oral administration to mice and rats. A xenograft breast cancer mouse model was chosen to delineate and evaluate *in vivo* the chemopreventive properties of lunasin and BBI separately and to elucidate the carcinogenesis pathways involved in breast cancer that are affected by these peptides.

## Results

### Lunasin Is a Bioactive Ingredient of BBIC

**Figure 1:**
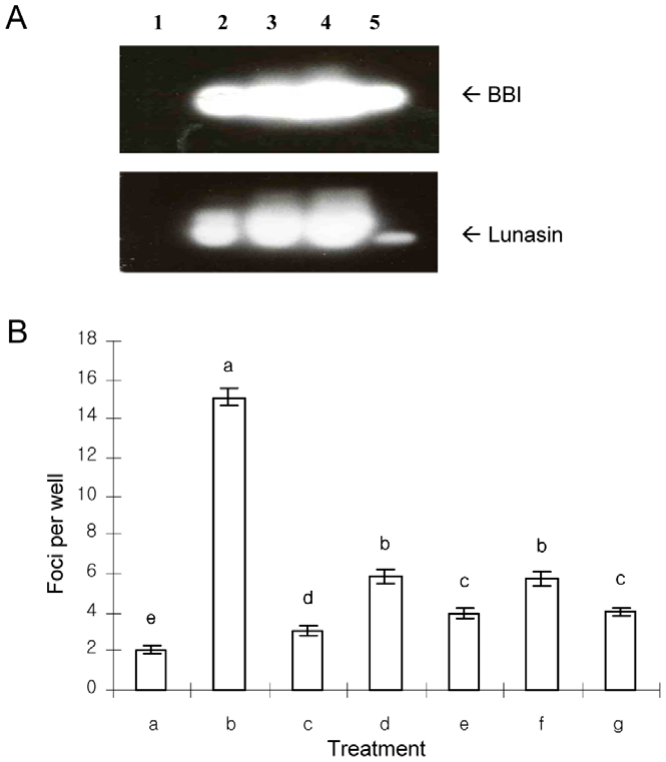
Lunasin is the main bioactive component in BBIC. **A**: Western-Blot of BBIC using antibodies specific for BBI and lunasin. (1) Marker, (2) 6.25 µg protein, (3) 12 µg protein, (4) 25 µg protein, (5) Standard BBI (3 µg) or standard lunasin (200 ng). **B**: Foci formation inhibitory activity in DMBA-induced NIH3T3 cells. (a) Vehicle-treated cells (b) positive control or DMBA-treated cells, (c) BBIC (100 nM lunasin +1160 nM BBI), (d) BBI (100 nM) from BBIC, (e) Lunasin (100 nM) from BBIC, (f) Standard BBI (100 nM), (g) Synthetic lunasin (100 nM). Bars with different lower case letter designations are statistically significantly different from each other (*P*<0.05, n = 6).

To determine the composition of BBIC, this preparation was subjected to SDS-PAGE and Western-Blot to identify lunasin and BBI. The results show that both peptides are present in BBIC at concentrations of 360 and 74.4 ng/µg protein, respectively (Figure 1A). The two represent about 44% of total protein of the BBIC, indicating that other proteins are present and may contribute to the properties attributed to this preparation.

Foci formation inhibitory activity of BBIC containing 1160 nM BBI and 100 nM lunasin was analyzed. BBIC suppressed foci formation by 80% in 7,12-dimethylbenz[a]anthracene (DMBA)-induced NIH3T3 cells, compared to vehicle-treated cells (Figure 1B**–**C). Lunasin and BBI were individually purified from the BBIC and their activities analyzed. 100 nM of pure lunasin isolated from BBIC reduced foci formation by 73% (Figure 1B**–**E), which is identical to that observed with 100 nM synthetic lunasin (Figure 1B**–**G). Pure BBI isolated from the BBIC at concentration of 100 nM decreases foci formation by 60% (Figure 1B**–**D). Thus, lunasin was more effective than BBI by 18% on an equimolar basis, but BBI also has a chemopreventive effect of its own, most likely due to inhibition of proteolytic processes involved in carcinogenesis.

### Soy Protease Inhibitors Protect Lunasin from *In Vitro* Digestion

**Figure 2:**
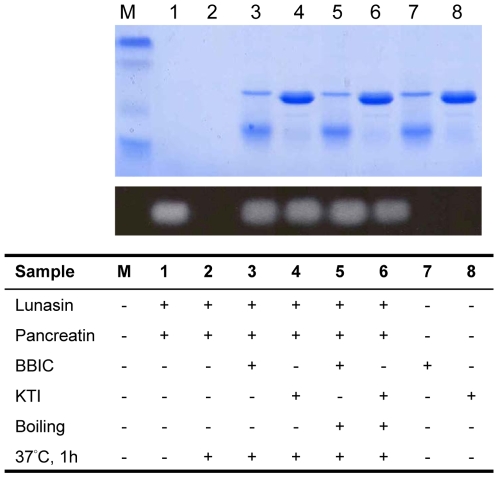
Soy-derived BBI and KTI protect synthetic lunasin from *in vitro* digestion. The upper, lighter bands are Coomassie Blue protein stains and the lower, darker bands are Western blots. (M) Markers, (1) Lunasin (600 ng) + pancreatin (600 ng) incubated for 0 h at 37°C, (2) Lunasin (600 ng) + pancreatin (600 ng) incubated for 1 h at 37°C, (3) Lunasin (600 ng) + BBI (18,000 ng, unboiled) + pancreatin (600 ng) incubated for 1 h at 37°C, (4). Lunasin (600 ng) + KTI (18,000 ng, unboiled) + pancreatin (600 ng) incubated for 1 h at 37°C, (5) Lunasin (600 ng) + BBI (18,000 ng, boiled) + pancreatin (600 ng) incubated for 1 h at 37°C, (6) Lunasin (600 ng)+KTI (18,000 ng, boiled) + pancreatin (600 ng) incubated for 1 h at 37°C, (7) BBI (unboiled), (8) KTI (unboiled).

In order to establish the role of the different protease inhibitors contained in soybean, such as BBI and Kunitz Trypsin Inhibitor (KTI), synthetic lunasin was subjected to an *in vitro* digestion process with pancreatin. Lunasin was incubated with this preparation in the absence and presence of BBI and KTI and evaluated by Western-Blot. The pattern shows (Figure 2) that both protease inhibitors protect lunasin from digestion, even after being denatured by heat treatment. Approximately 93% and 97% of lunasin remain intact after the digestion process in the presence of unboiled BBI and KTI, respectively. Similar percentages of lunasin (98% and 84%) remained intact when this peptide was incubated with pancreatin in the presence of boiled BBI and KTI, respectively. Lunasin resists heat treatment [17], and is present in different processed soybean products that also contain BBI [18]. All these results indicate that lunasin would be bioavailable after ingestion of these products due to the protective role of BBI and other soy protease inhibitors.

### Lunasin Is Bioavailable When Orally Administered to Mice and Rats

**Figure 3:**
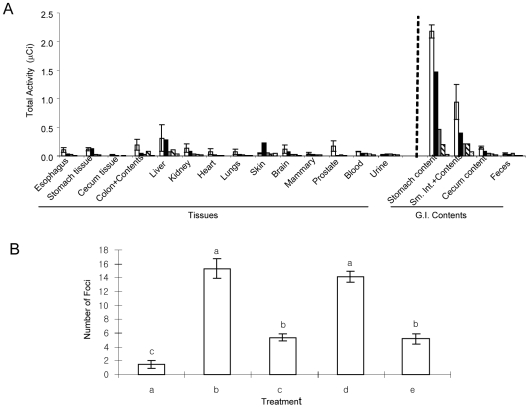
Lunasin is absorbed through gastrointestinal barrier and reach the target tissues and organs in an intact and bioactive state. **A**: Distribution of ^3^H-lunasin activity in various tissues and gastrointestinal contents of mice after (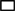) 3 h, (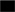) 6 h, (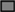) 9 h, (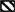) 12 h and (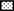) 24 h of oral administration of LES. Error bars shown for 3 h-results. **B**: Foci formation inhibitory activity in DMBA-induced NIH3T3 cells. (a) Vehicle-treated cells, (b) DMBA-treated cells, (c) synthetic lunasin, (d) lunasin extracted from liver of rats fed control diet, (e) lunasin extracted from liver of rats fed LES diet. Bars with different lower case letter designations are statistically significantly different from each other (*P*<0.05, n = 6).

Mice and rats were used to determine whether orally ingested lunasin survives digestion, ends up in the tissues and remains intact and bioactive as measured by an *in vivo* assay. In the first set of experiments, CD-1 mice received ^3^H-labelled synthetic lunasin mixed with lunasin-enriched soy (LES) by gavage. Lunasin is absorbed and distributed in various collected tissues, including those that are targets for the most common cancers, such as lung, mammary gland and prostate (Figure 3A). It is noteworthy that lunasin is able to cross the blood-brain barrier, reaching the brain. At 3 hrs post-gavage, approximately 30% of the total oral dose of lunasin is absorbed (Figure S1).

Since the ^3^H-radioactivity would not show the molecular size and bioactivity of lunasin in the tissues, rats were fed dietary LES for 4 weeks, the blood and liver were collected and lunasin was extracted, purified, and analyzed by Western-blot. Lunasin is present in the blood of LES-fed rats as a monomer at a concentration of 17.5 ng/ml. However, lunasin isolated from the liver of LES-fed rats exists as a dimer at a concentration of 3.1 µg/g of liver (Figure S2). Lunasin (1 µM) extracted from the liver of LES-fed rats suppresses foci formation as effectively as an equimolar amount of synthetic lunasin (Figure 3B). In contrast, the control protein band extracted from LES-unfed rats is ineffective in suppressing foci formation.

### Lunasin Reduces Tumor Incidence and Generation

**Figure 4:**
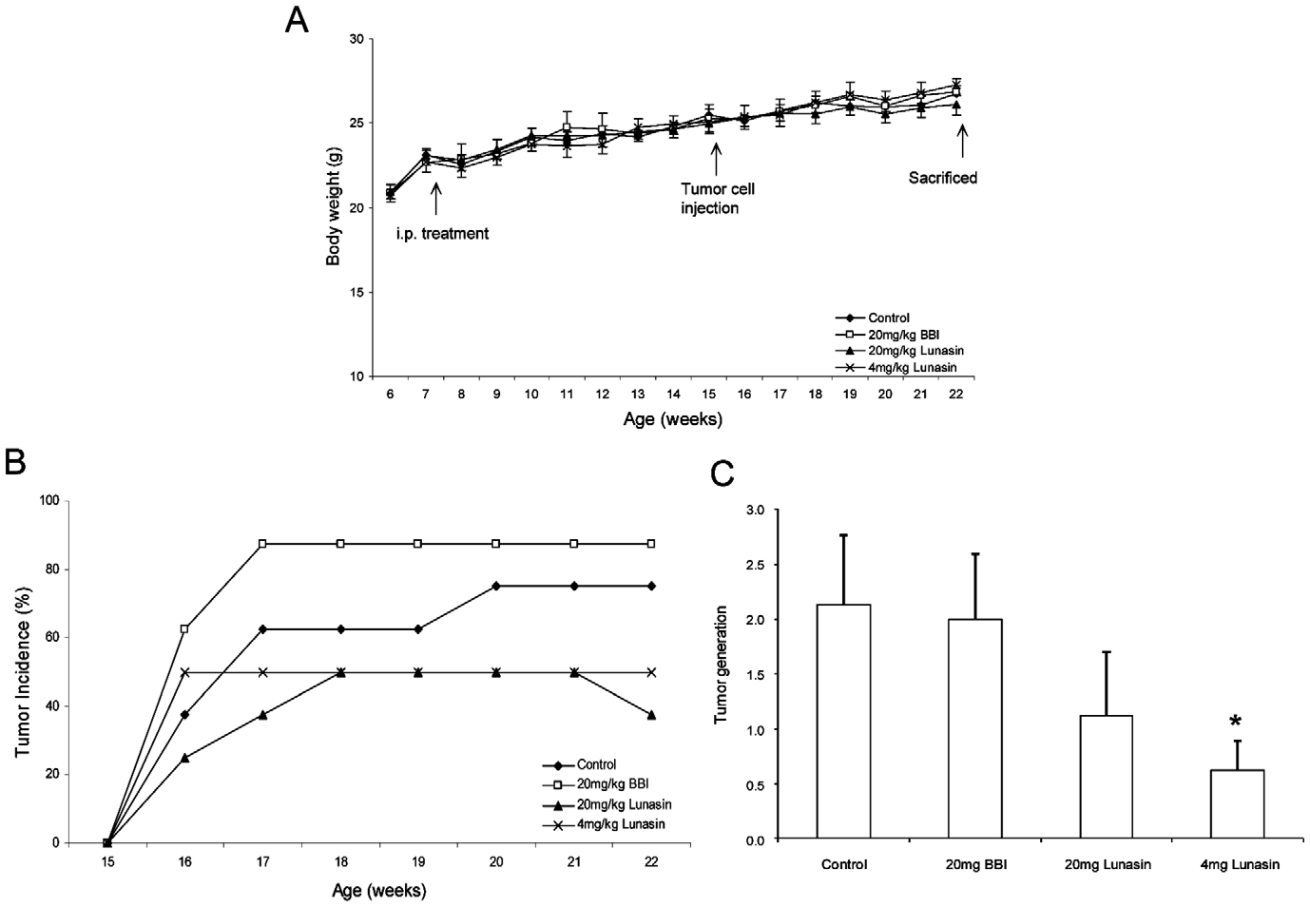
Lunasin reduces breast tumor development in MDA-MB-231 tumor xenografts in athymic nude mice. **A**: Body weight of mice treated with (♦) PBS (control), (□) 20 mg BBI/kg body weight, (▴) 20 mg lunasin/kg body weight, and (×) 4 mg lunasin/kg body weight, and injected with 1×10^7^ MDA-MB-231 cells in the flank of 15-week-old nude mice. No significant differences were observed among four groups. **B**: Tumor incidence in mice treated with PBS, BBI and two doses of lunasin. Lunasin reduced number of mice that showed tumors and delayed the appearance of these breast tumors. **C**: Tumor generation in mice treated with PBS, BBI and two doses of lunasin. Low dose of lunasin (4 mg/kg body weight) significantly reduced tumor generation compared to control group (*P = 0.029). Data are shown: mean ± SEM (n = 8).

To examine the *in vivo* effect of lunasin and BBI in breast cancer cells, MDA-MB-231 cells were implanted subcutaneously into nude mice after 2 months intraperitoneal (i.p.) injection of lunasin and BBI. No significant differences in body weights were observed among the four groups (Figure 4A), suggesting that lunasin or BBI treatments has no side effects on the general health condition. These results are consistent with studies carried out by Johnson and coworkers [19] who reported no significant effects on body weight after daily gavage of BBIC (500∼2000 mg/kg/day) for six months.

Breast cancer incidence of mice is shown in Figure 4B. At seven weeks post-cells injection, 75% and 88% of mice in control and BBI-groups, respectively showed tumors. In contrast, tumor incidence was 38% and 50% in mice treated with 20 mg/kg and 4 mg/kg lunasin, respectively. Thus, compared with the control, the tumor incidence was 49% and 33% lower in the lunasin-treated groups respectively. Compared with the BBI group, the tumor incidence was 57% lower in the 20 mg/kg and 43% lower in the 4 mg/kg lunasin treated group. Mice treated with lunasin also showed a delay in the appearance of tumors. Moreover, tumor generation, was reduced in the two groups of mice treated with lunasin, being significantly lower in mice treated with the lowest dose of lunasin, 70% and 69% lower relative to control and BBI group respectively (*P* = 0.029 vs control) (Figure 4C).

**Figure 5:**
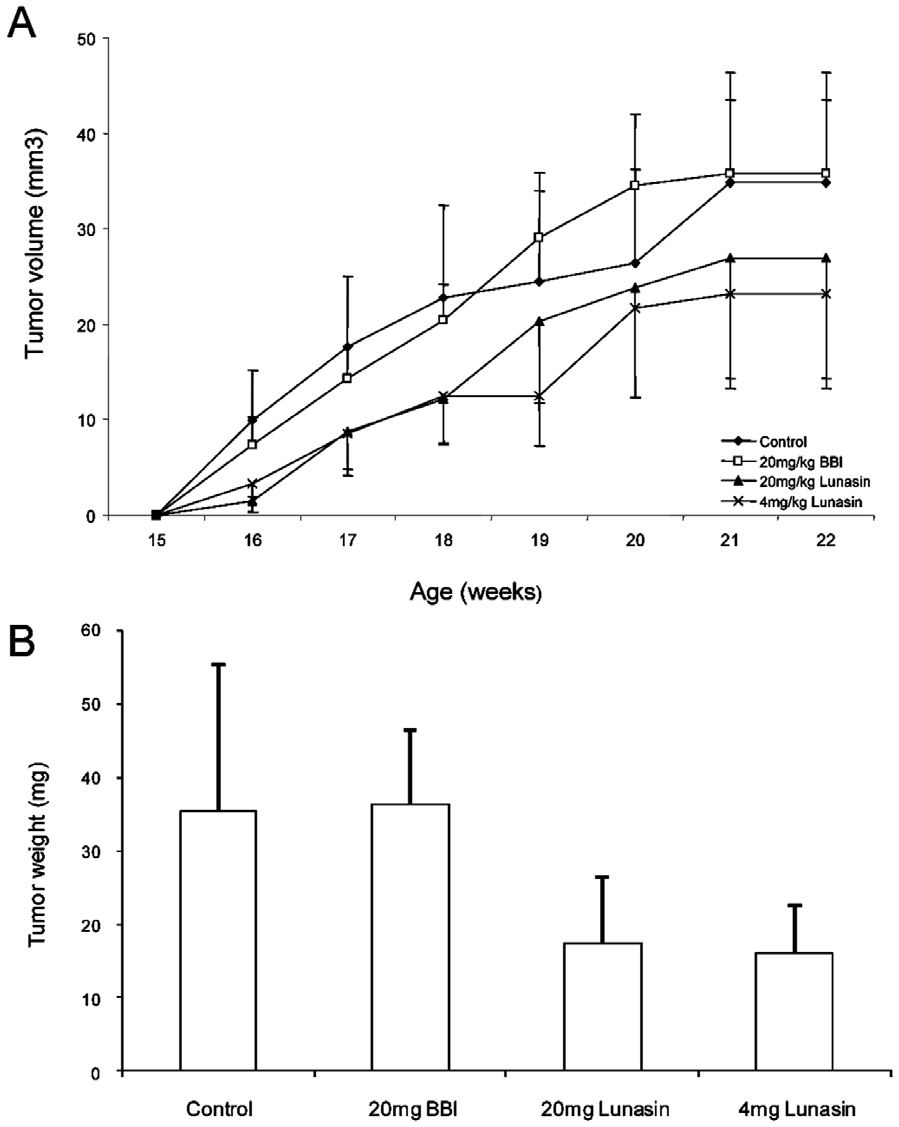
Effects of BBI and lunasin on the growth of MDA-MB-231 tumors in athymic nude mice. **A**: Tumor volume (mm^3^) of breast tumors induced by the administration of (♦) PBS (control), (□) 20 mg BBI/kg body weight, (▴) 20 mg lunasin/kg body weight and (×) 4 mg/kg of lunasin. **B**: Breast tumors weight induced by PBS (control), 20 mg BBI/kg body weight, 20 mg lunasin/kg body weight (*P* = 0.2134 vs control group and 0.0909 vs BBI group) and 4 mg lunasin/kg body weight (*P* = 0.1880 vs control group and 0.0569 vs BBI-group). Data are shown mean ± SEM (n = 8).

Growth rate and final size of the tumors differed among the four groups. Significant tumors appeared in control mice after cells injection whereas the sizes of tumors in mice treated with 20 mg/kg and 4 mg/kg lunasin were decreased by 23% and 34%, respectively (Figure 5A). At the end of the experiment, the weight of tumors was also lower in mice treated with both doses of lunasin compared to control-group (*P* = 0.2134, 0.1880, respectively) and BBI-group (*P* = 0.0909, 0.0569, respectively) (Figure 5B).

**Figure 6:**
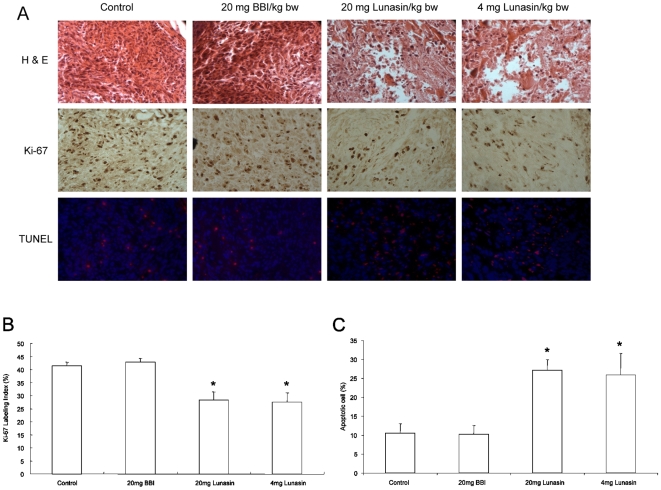
Histological features of MDA-MB-231 tumors treated with PBS (control), BBI and lunasin in athymic nude mice (400× magnification). **A**: Tumors were collected at 7 week after breast cancer cells injection, and processed for H&E staining. Representative pictures are shown for H&E-stained sections (upper column), immunohistochemical staining for Ki-67 (middle column) and in situ TUNEL apoptotic indices (bottom column). **B**: Percentage of Ki-67 expression in tumors of mice. Ki-67 labeling revealed significantly lower levels of proliferating cells in lunasin groups versus control group (*P = 0.0062 in high lunasin group; *P = 0.0158 in low lunasin group). **C**: Percentage of apoptotic cells in tumors of mice measured with the TUNEL assay. Staining revealed significantly higher levels of apoptotic tumor cells in lunasin groups versus control group. (*P = 0.0088 in high lunasin group; 0.0141 in low lunasin group). Data are shown mean ± SEM.

Palpable and non-palpable mammary tumors were collected and subjected to histological analysis and immunostaining. After H&E staining (Figure 6A), tumor sections of lunasin-treated groups showed tumor destruction areas replaced by apoptotic and necrotic cells that were not apparent in the control and BBI-treated groups. To establish greater specificity for the antiproliferative response, tumor sections were analyzed by immunohistochemistry for Ki-67 expression, an indicator of cell proliferation [20]. Lunasin treatment at 20 mg/kg and 4 mg/kg reduced Ki-67 expression by 34% (*P* = 0.0062) and 30% (*P* = 0.0158), respectively, compared with the control group (Figure 6B). However, there was no significant difference between the Ki-67 expression of control and BBI-groups. In situ TUNEL assay was performed to evaluate the apoptotic effect of lunasin and BBI treatments (Figure 6C). TUNEL-positive apoptotic cells were found in tumors from animals treated with both doses of lunasin, revealing a significantly increased apoptosis in these treatment groups compared to the control group (*P* = 0.0088, 0.0141, respectively). Few TUNEL-positive cells were found in tumors from animals in the BBI-group. These suggest that tumors from mice treated with lunasin have significantly lower rate of proliferation and higher apoptosis index compared to control and BBI groups.

## Discussion

The high prevalence of breast cancer has provided a strong rationale for identifying new compounds for use as preventive and/or therapeutic agents. Epidemiological studies, animal experiments and human trials have shown evidence that people consuming a soy-rich diet have lower incidence and mortality from breast cancer, leading to investigations on different compounds from soy that provide protection against breast cancer [21], [22]. Daily intake of these anticancer compounds could be compared to a preventive, non-toxic version of chemotherapy that is harmless to the physiology of normal tissue and stops microtumours [23], [24]. To evaluate the potential risks and benefits of phytochemicals to human health, understanding of the physiological behavior of these compounds following oral ingestion as well as of their absorption, distribution, metabolism, and excretion is needed [15].

The soybean BBIC is largely a preparation containing the protease inhibitor BBI that has been used to demonstrate the chemopreventive properties of this peptide avoiding the high cost of its purification. Several studies have reported the chemopreventive role of the BBIC against different types of cancer induced by chemical carcinogens and radiations [14], [25]. These studies have assumed that BBI is the main bioactive compound responsible for BBIC's chemopreventive activity, without evaluating the contribution of other minor peptides. Our results show that BBIC contains both lunasin and BBI representing 44% of total protein. The *in vitro* foci formation inhibitory activity assay shows that lunasin exerts 18% higher activity in DMBA-induced NIH3T3 cells than BBI on an equimolar basis. However, it is clear that the protease inhibitory activities of BBI and KTI are essential in protecting lunasin from *in vitro* digestion with pancreatin. *In vivo* experiments, carried out with mice and rats fed LES, have demonstrated that lunasin, orally ingested, resists digestion, gets absorbed and reaches the target tissues and organs in an intact and bioactive state. Recently, it has been demonstrated that lunasin is bioavailable in humans fed soy protein products, an important requirement for its anticancer potential [26]. Thus, the protective roles of BBI and KTI and perhaps other naturally occurring protease play a major role in making lunasin available in soy protein to exert its chemopreventive properties. Bioavailability after oral administration of a chemopreventive agent is crucial to understanding the various *in vivo* mechanisms responsible for its cancer preventive activity [27].

In order to delineate the individual *in vivo* preventive properties against breast cancer of lunasin and BBI, each peptide was injected separately into nude mice before and after injection of MDA-MB-231 cells. In our study, one dose of BBI and two doses of lunasin were assessed. The low dose (4 mg/kg body weight) of lunasin corresponds to the daily intake of soy protein considered by FDA to reduce cardiovascular disease, and also in accordance to the results of our present study showing that 30% of ingested lunasin reaches the target tissues. The use of the higher dose (20 mg/kg body weight) is based on the finding that i.p. administration of BBI at this dose significantly suppresses effect on 3-methylcholanthrene-induced lung tumors in A/J mice [28]. Both doses of these peptides appear to be well tolerated by the mice as evidenced by lack of change in body weight during the 15-week treatment period. Mice treated with lunasin show reduction in breast tumor incidence and delay in the appearance of tumors. Tumor generation is also significantly inhibited at the lower dose of lunasin. Moreover, the volume and weight of tumors generated in lunasin-treated groups are lower compared with control and BBI-groups. No effects on breast tumor development are observed when BBI was used in MDA-MB-231 xenograft mice.

In order to better understand the specific mechanisms by which lunasin exerts its effects on MDA-MB-231 breast tumors, biomarkers of cell proliferation and apoptosis which are good indicators of tumor size in xenograft model were evaluated [29], [30]. Histological staining of sections obtained from lunasin-treated tumors shows that tumor destruction areas are replaced by apoptotic and necrotic cells. Lunasin treatment also results in a significant reduction of cell proliferation and induction of apoptosis in the MDA-MB-231 tumors. These effects are not observed in tumors from BBI-treated mice.

Chemopreventive properties of lunasin have been demonstrated *in vivo* in previous studies. In the first animal model, lunasin applied topically at 250 mg/week suppresses skin papilloma formation in SENCAR mice treated with DMBA and tetradecanoylphorbol-13-acetate by 70% compared with the control. Tumor multiplicity is also reduced and the appearance is delayed by 2 weeks in mice treated with lunasin relative to the control [10]. This is consistent with another observation that lunasin slows down epidermal cell proliferation in mouse skin in the absence and presence of DMBA using a ^2^H_2_O labeling method to measure cell proliferation *in vivo* [31]. All these support our findings that lunasin acts as a cancer preventive agent *in vivo*.

Kennedy has demonstrated that BBI has significant cancer chemopreventive activity in both *in vitro* and *in vivo* bioassay systems [25]. Although BBI has a broad spectrum of cancer-protective activities, its effects on breast cancer remains limited. BBI has been previously analyzed for its bioactivity against other types of cancer, such as oral mucosa, colon, and lung cancer [28], [32], [33]. However, no *in vivo* studies reporting the effect of BBI as breast cancer preventive peptide have been published. Nevertheless, there are some *in vitro* studies showing that BBI decreased estrogen dependent human breast cancer cell growth [34]–[36]. Wan and coworkers [37] demonstrated the growth inhibitory properties of BBI in human prostate cancer xenografts in nude mice. Our results using the xenograft model where lunasin and BBI were evaluated separately show that BBI exhibits very little chemopreventive effect against breast cancer. More studies are needed to determine if tumor cell type and its specific carcinogenesis pathways may be determinants of the cancer chemopreventive properties of BBI.

In summary, our findings show that lunasin and BBI are the two main bioactive ingredients of BBIC. In foci formation assay, lunasin is about 18% more effective than BBI. However, the *in vivo* xenograft model shows that while lunasin exhibit substantial chemopreventive and therapeutic effects, BBI shows very little. We propose that to explain the observed chemopreventive properties of soy and other seeds containing lunasin, naturally occurring protease inhibitors such as BBI and KTI, mainly protect lunasin from digestion, making it bioavailable. This theme of complementarity of naturally occurring molecules in bringing about health benefits to humans is likely quite common in nature and speaks for eating whole foods rather than isolated components.

## Materials and Methods

### Purification of Lunasin and BBI from BBIC

To prepare the BBIC, 50 g finely-ground soybeans were extracted with 1250 ml of hexane at 4°C for 24 hrs and then re-extracted with 1250 ml of 60% ethanol at 55∼60°C for 1 h. After extraction, pH of the solution was adjusted to 5.3, and the BBIC was precipitated with 2500 ml of acetone for 15 min. The precipitate was dissolved in 100 ml of distilled water, dialyzed at 4°C for 24 hrs, and freeze-dried.

Separation of lunasin and BBI from the BBIC was carried out by ion exchange chromatography. The column (AG MP-1M, 5.0×50 cm, Bio-Rad Laboratories, Hercules, CA, USA) was equilibrated with 0.1 M sodium phosphate buffer saline (PBS, pH 7.0). Five hundred mg of BBIC dissolved in 0.1 M PBS were applied into the column and chromatographic separations were carried out with various concentration of NaCl in PBS at 4°C at a flow rate of 30 ml/h. The different collected fractions were subjected to Western-Blot for detection of lunasin and BBI as described previously [8]. Briefly, SDS-PAGE was carried out using 16.5% tris-tricine gels (Bio-Rad). The proteins were transblotted onto nitrocellulose membrane and blocked in 5% nonfat dry milk dissolved in Tris-buffered saline 1% Tween 20 (TBS-1T). The membrane was washed with TBS-1T and incubated with lunasin polyclonal antibody (Zymed, Inc., South San Francisco, CA, USA), or monoclonal BBI antibody, kindly provided by Dr. David Brandon (USDA, WRRL, Albany, CA, USA). After washing, the membrane was incubated for 1 h with horseradish peroxidase conjugated secondary antibodies (Santa Cruz Biotechnology, Santa Cruz, CA, USA). The membrane was detected using the detection agent (Amersham Biosciences, Piscataway, NJ, USA) and immediately developed using 667 Polaroid films. The intensities of the bands were quantified using the software *Un-SCAN-IT gel* version 5.1 (Silk Scientific, Inc. Orem, UT, USA). Lunasin and BBI contents were calculated by comparing the band intensities with those of known standards lunasin and BBI run under the same conditions.

### Foci Formation Assay

The foci formation assay was performed according to Reznikoff et al [38]. NIH3T3 cells were obtained from American Type Culture Collection (ATCC) and cultured in RPMI 1640 medium supplemented with 10% fetal bovine serum (FBS) (Invitrogen, Carlsbad, CA, USA). Cells were plated at a density of 500 cells/well in 12-well plates, incubated overnight at 37°C and treated with lunasin or BBI for 4 hrs. Then, cells were treated with 1.5 µg/ml DMBA for 20 hrs. After washing with PBS, fresh medium was added. Peptides were added and medium was changed every week for 6 weeks. At the end of experiment, cells were washed with 0.9% NaCl, fixed with methanol, stained with Giemsa, and scored for transformed foci. The negative control was a set of cells receiving no DMBA, while the positive control consisted of cells induced by DMBA but without treatment.

### *In Vitro* and *In Vivo* Bioavailability Studies

*In vitro* digestion of lunasin was carried out following the method published in the United States Pharmacopeia [39]. Synthetic lunasin (American Peptide Company, Inc. Sunnyvale, CA, USA) (5 µg) was incubated with the protease inhibitors BBI or KTI (Sigma, St. Louis, MO, USA) for 30 min at 25°C. Pancreatin from porcine pancreas (Sigma) was added (enzyme∶protein ratio of 1∶10) and solution was incubated at 37°C for 1 h. Tris-tricine sample buffer was added at the end of the reaction and immediately stopped by placing the tubes in a boiling water bath for 5 min. The digests were analyzed by SDS-PAGE and Western-Blot following the protocol previously described.

Bioavailability studies in mice were done at North View Pacific Laboratories (Hercules, CA, USA), according with the Animal Care and Use Committee (ACUC) human guidelines. CD-1 mice (3-5 weeks old) were purchased from Jackson Laboratories (Bar Harbor, ME, USA) and conditioned on AIN76A diet for one week. Forty mice (20 male and 20 female) were fasted for 8 hrs before oral administration by gavage. Mice were separated into two groups, a control group receiving 240 mg of LES formulation and a treatment group receiving 240 mg of LES formulation plus 8 µCi of ^3^H-lunasin (SibTech, Inc. Brookfield, CT, USA) in 0.1 mL of 10% sucrose solution. Four mice of each group were sacrificed at 3, 6, 9, 12 and 24 hrs after oral administration. The tissue samples were collected and prepared for scintillation counting by solubilizing in TS-2 tissue solubilizer (Research Products International Corp., Chicago, IL, USA) and addition of 15% benzoyl peroxide. Each sample was read in 5 ml of HionicFluor cocktail mix using a 1600TR liquid scintillation counter (Packard Inst. Meriden CT, USA).

Sprague-Dawley Rats (Female, 5-weeks old) were purchased from Central Laboratory Animal Inc. (Korea), and experiments were done according with guidelines of the Animal Center at Andong National University (Andong, Korea). Five rats were fed *ad libitum* with LES and potato starch (0.25% of the diet) for 4 weeks, and then sacrificed. Livers and serum were collected and freeze-dried immediately. One gram of the freeze-dried tissues was extracted with 0.1 M PBS (pH 7.0) (buffer∶tissue ratio (v/w) = 1∶3) at 4°C for 24 hrs, and then centrifuged at 15000 rpm for 1 h. The supernatant protein was further purified by ion exchange chromatography on Biogel resin AG 1-X4 (BioRad), as described by Jeong et al. [40]. Lunasin was further purified by injecting 20 µl of filtrate into the HPLC system equipped with a C18 column (Proteo 90A, Phenomenex Co., Torrance, CA, USA) equilibrated at ambient temperature and an UV detector (295 nm) stabilized with the mobile phase (acetonitrile∶water = 4∶6), at a flow rate of 2.5 ml/min for 15 min. Lunasin was identified by comparison with the retention time of standard synthetic lunasin peak. The lunasin fraction was collected, freeze dried and analyzed by SDS-PAGE and Western-Blot as described above.

### Breast Cancer Prevention in a Xenograft Mouse Model

Human MDA-MB-231 breast cancer cells were obtained from ATCC and grown in Leibovitz's L-15 Medium supplemented with 10% FBS, 100 unit/ml penicillin, and 100 µg/ml streptomycin (Invitrogen), in a humidified atmosphere at 37°C.

Mice protocol was approved by ACUC (University of California, Berkeley, CA, USA). Thirty-two six weeks-old athymic NCr-nu/nu mice (National Cancer Institute, Frederick, MD, USA) were acclimatized for 1 week and randomly divided into four groups (n = 8): control group (receiving PBS), BBI-group (receiving BBI at 20 mg/kg body weight), and two lunasin-groups (receiving synthetic lunasin [Chengdu KaiJie Bio-Pharmaceutical Co., Chengdu, P.R. China] at 20 mg/kg body weight and 4 mg/kg body weight). After two months of i.p. treatment three times a week, mice were injected subcutaneously in the right flank with 1×10^7^ MDA-MB-231 cells suspended in 0.1 ml of Matrigel Basement Membrane Matrix (Becton Dickinson, Bedford, MA, USA). Tumor growth was monitored by palpation, and the onset when tumors were detectable was noted. Tumor size was measured with calipers, and tumor volume was calculated assuming the shape as ellipsoid. Tumor incidence in percentage was calculated as the number of tumor-bearing mice divided by the total number of mice in each group, whereas tumor generation was calculated as the total number of tumors divided by total number of mice in each group [10]. The volume of the tumor was determined using the following formula: tumor volume = length × width^2^ ×0.523 [41].

### Histological Analysis

At the end of the experiment, animals were sacrificed, and tumors were dissected and weighed. Individual tumors were split for fixation in the Shando Glyo-Fixx™ (Thermo Scientific, Pittsburgh, PA, USA) for histological examination. The paraffin-embedded sections were stained with hematoxylin and eosin (H&E).

### Immunohistochemical Staining for Ki-67 Labeling Index

Sections were evaluated for tumor cell cytology, growth pattern, necrosis and apoptosis. The 5 µm sections were deparaffinized and rehydrated and antigens were retrieved using citrate buffer (pH 6.0) before endogenous peroxidase activity was blocked. Proliferation was shown using an antibody against Ki-67 (MIB-1; DakoCytomation, Glostrup, Denmark, dilution 1∶75). Antigen expression was expressed using streptavidin conjugated to horseradish peroxidase and visualized by diaminobenzidine chromogen reagent (DakoCytomation). All immunohistochemistry slides were examined by light microscopy (Axiophot 381, Zeiss, Germany). Ki-67 labeling index was calculated as percentage of positive cells over total number of cells counted in microscope at 400× magnification [30].

### *In Situ* TUNEL Assay for Apoptosis

In situ terminal deoxynucleotidyl transferase-mediated dUTP nick end labeling (TUNEL) assay was performed using ApopTag Detection Kit (Millipore, USA & Canada) and ran based on the manufacturer's protocol. Briefly, deparaffinized and rehydrated sections were pretreated with 20 µg/mL proteinase K for 15 min and incubated with terminal transferase and digoxigenin dUTP at 37°C for 1 h. After washing, sections were incubated with antidigoxigenin rhodamine (Millipore) and DAPI (Invitrogen), and slides were visualized by fluorescent microscopy (Axiophot 381). The apoptotic cells were calculated as percentage of positive cells over total number of cells counted in microscope at 400× magnification.

### Statistical Analysis

All data were analyzed from three independent experiments. Results were expressed as the mean ± standard deviation. The Duncan test and one-way analysis of variance (ANOVA) were used for multiple comparisons (SPSS software, version 12.0) of data obtained by *in vitro* experiments. Comparison of results obtained from animal experiments was performed using Student's t-test. A *P*-value less than 0.05 was considered statistically significant.

## Supporting Information

Figure S1 Recovery of ^3^H-lunasin dose (expressed in percentage) in (-▴-) total, (-•-) tissues, and (-o-) gastrointestinal contents of mice at 3, 6, 9, 12, and 24 hours post-gavage of lunasin-enriched soy (LES). CD-1 mice received 240 mg of LES formulation plus 8 µCi of ^3^H-lunasin (SibTech) in 0.1 ml of 10% sucrose solution. Before gavage, the mice were fasted for 8 hours and then sacrificed at 3, 6, 9, 12, and 24 hrs after oral administration.
(0.39 MB TIF)

Figure S2 Lunasin was extracted from blood and liver of rats fed control and lunasin-enriched soy (LES) diets for four weeks. Upper panels of the figure (A and C) correspond to gels stained with Coomassie Blue of blood and liver, respectively. Lower panels (B and D) correspond to Western blot analysis of blood and liver, respectively. 1: MW marker; 2: Blood and 2′ liver from rats fed control diet; 3: Blood and 3′ liver from rats fed control diet and purified by anion exchange-HPLC; 4: Blood and 4′ liver from LES-fed rats; 5: Blood and 5′ liver from LES-fed rats and purified by anion exchange-HPLC; L: Synthetic lunasin 165 nM. Lunasin contained in the blood of LES-fed rat was only detectable after purification process.
(2.09 MB TIF)
