# Supplementary figures and images for: Complementary Roles in Cancer Prevention: Protease Inhibitor Makes the Cancer Preventive Peptide Lunasin Bioavailable
Source: PLoS One. 2010 Jan 26;5(1):e8890. doi: 10.1371/journal.pone.0008890 (PMC2811193; doi:10.1371/journal.pone.0008890)

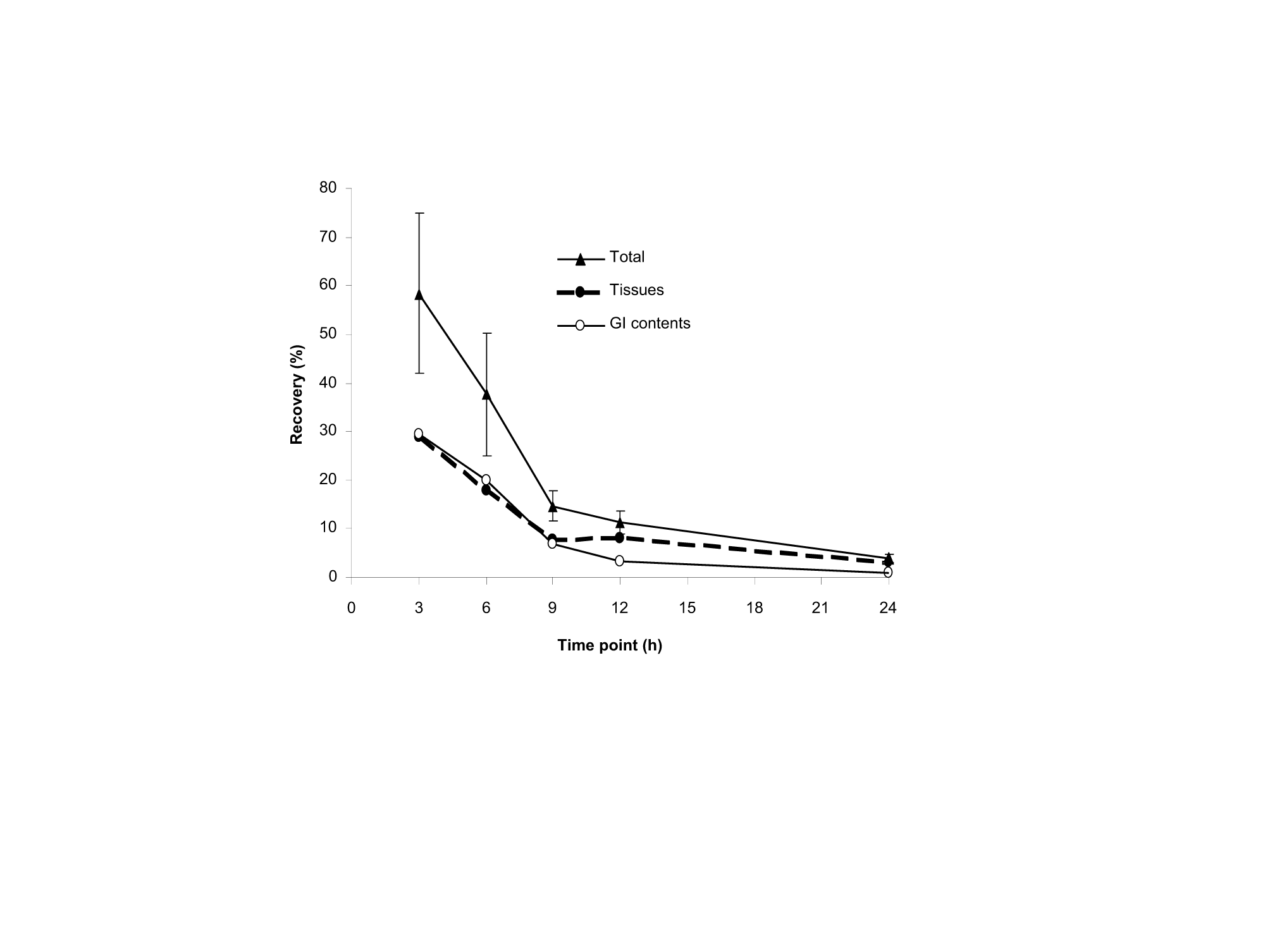

Supplement: Figure S1 — Recovery of 3H-lunasin dose (expressed in percentage) in (-▴-) total, (-•-) tissues, and (-o-) gastrointestinal contents of mice at 3, 6, 9, 12, and 24 hours post-gavage of lunasin-enriched soy (LES). CD-1 mice received 240 mg of LES formulation plus 8 µCi of 3H-lunasin (SibTech) in 0.1 ml of 10% sucrose solution. Before gavage, the mice were fasted for 8 hours and then sacrificed at 3, 6, 9, 12, and 24 hrs after oral administration. (0.39 MB TIF) [file pone.0008890.s001.tif]

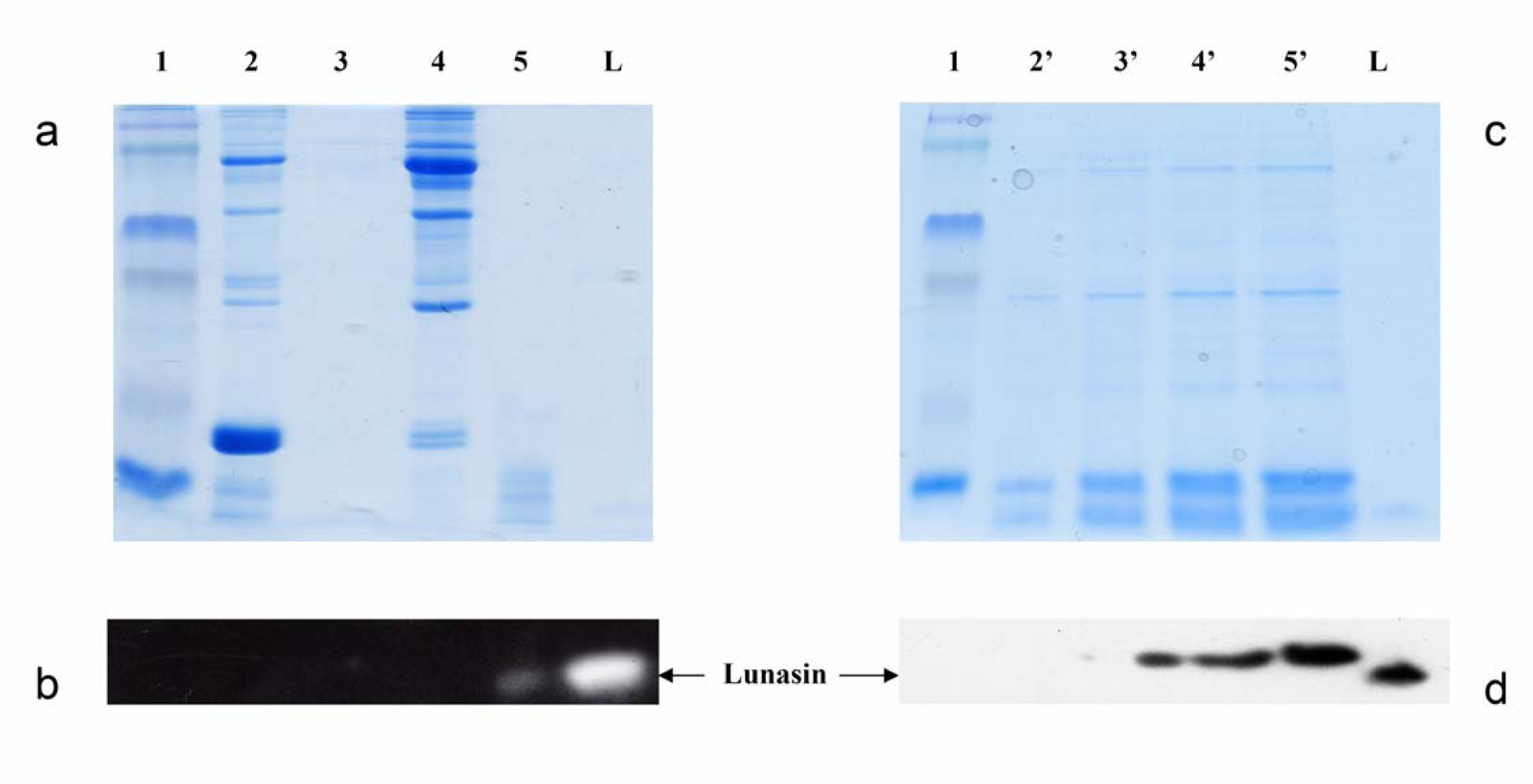

Supplement: Figure S2 — Lunasin was extracted from blood and liver of rats fed control and lunasin-enriched soy (LES) diets for four weeks. Upper panels of the figure (A and C) correspond to gels stained with Coomassie Blue of blood and liver, respectively. Lower panels (B and D) correspond to Western blot analysis of blood and liver, respectively. 1: MW marker; 2: Blood and 2′ liver from rats fed control diet; 3: Blood and 3′ liver from rats fed control diet and purified by anion exchange-HPLC; 4: Blood and 4′ liver from LES-fed rats; 5: Blood and 5′ liver from LES-fed rats and purified by anion exchange-HPLC; L: Synthetic lunasin 165 nM. Lunasin contained in the blood of LES-fed rat was only detectable after purification process. (2.09 MB TIF) [file pone.0008890.s002.tif]
